# Multifunctional Water Sensors for pH, ORP, and Conductivity Using Only Microfabricated Platinum Electrodes

**DOI:** 10.3390/s17071655

**Published:** 2017-07-19

**Authors:** Wen-Chi Lin, Klaus Brondum, Charles W. Monroe, Mark A. Burns

**Affiliations:** 1 Department of Chemical Engineering, University of Michigan, Ann Arbor, MI 48109, USA; wclin@umich.edu; 2 Masco Corporation, Taylor, MI 48180, USA; klaus_brondum@mascohq.com; 3 Department of Engineering Science, University of Oxford, Oxford OX1 3PJ, UK; charles.monroe@eng.ox.ac.uk; 4 Department of Biomedical Engineering, University of Michigan, Ann Arbor, MI 48109, USA

**Keywords:** micro-fabrication, ORP sensor, pH sensor, conductivity sensor, water safety

## Abstract

Monitoring of the pH, oxidation-reduction-potential (ORP), and conductivity of aqueous samples is typically performed using multiple sensors. To minimize the size and cost of these sensors for practical applications, we have investigated the use of a single sensor constructed with only bare platinum electrodes deposited on a glass substrate. The sensor can measure pH from 4 to 10 while simultaneously measuring ORP from 150 to 800 mV. The device can also measure conductivity up to 8000 μS/cm in the range of 10 °C to 50 °C, and all these measurements can be made even if the water samples contain common ions found in residential water. The sensor is inexpensive (i.e., ~$0.10/unit) and has a sensing area below 1 mm^2^, suggesting that the unit is cost-efficient, robust, and widely applicable, including in microfluidic systems.

## 1. Introduction

Water monitoring in both developing [1] and developed countries [2] is a research area of considerable importance. Water quality analysis typically involves the measurement of several variables simultaneously, including conductivity, pH, and oxidation-reduction-potential (ORP)—each of which will have a different acceptable range depending on the application. For instance, conductivity represents the total dissolved solids (TDS) in water, and according to the United States Environmental Protection Agency (EPA), the TDS of drinking water should be less than 500 mg/L, which translates to a conductivity of around 800 µS/cm. Swimming pools, on the other hand, are not considered dangerous until their conductivity exceeds 4000 µS/cm. Besides conductivity, the pH and ORP of water should also be monitored. According to the EPA, the ORP of drinking water should be around 250 mV and the pH should be 6.5 to 8.5. Conversely, the ORP of swimming pools should be above 650 mV [3]. Abnormal values of pH or ORP in such applications can indicate that pollutants have contaminated the supply and that the situation is not safe. There are many other examples (e.g., aqueous process flow streams) for which accurate measurements would be beneficial. However, measuring each variable typically requires a separate meter that costs $100 or more, and the probes for each meter are about the size of a pen or larger. The total cost and size of water safety meters thus are relatively high and are not practical in chip-based applications.

The development of scalable, stable, and versatile integrated sensors for water quality analysis that can be widely applied is needed. Water contamination commonly occurs downstream of the water treatment plants, indicating that end-point monitoring at home is crucial for users’ safety. A small and affordable sensor with a long lifetime is thus needed. Though micro-conductivity sensors have been developed [4], the development of pH and ORP sensors is limited by the instability of solid-state reference electrodes (SSREs). Previous studies have focused on developing stable Ag/AgCl SSREs, but Ag/AgCl SSREs have limited lifetimes because the deposited AgCl electrodes eventually dissolve into the test solutions [5]. The lifetime of the SSREs can be extended by using barrier layers over the deposited AgCl to lower the AgCl dissolution rate [6,7,8]. Substantial research has also focused on the identification of new stable SSRE chemistries [9,10,11], but most of them still require membranes to block the interfering ions from the solution. The other challenging component is the pH sensing system, because pH sensors have to be insensitive to the environment except for the existence of hydronium ions. Some researchers focus on pH-sensitive antimony [12] or Ir/IrO_2_ electrodes [9,13], and other researchers focus on silicon nanowire pH sensors [14,15,16]. Though trade-offs between sensitivity and stability of silicon nanowires is commonly observed [15], silicone nanowires draw great attention for their fast response and high sensitivity [16]. Despite their great accuracy, these pH-sensing systems still need a stable SSRE.

In this paper, we present a sensing method that requires only three simple platinum electrodes to detect the conductivity, ORP, and pH of aqueous solutions. The electrodes on our sensor require no membranes or nanostructured material, and contain only a single layer of physical vapor deposition (PVD) Ti/Pt. The relatively simple fabrication suggests low production costs, and the units can be easily integrated with other devices. The small area of the three electrodes (<1 mm^2^) and robust materials suggest that this sensor is suitable to be embedded at the end-points in the service lines. Since scalable and cost-efficient end-point monitoring with low maintenance is crucial for drinking water safety, the proposed method is a potential approach for water monitoring.

The main advance of this experimental work is the simplicity of construction coupled with the integration of multiple sensors in a robust format, and not the development of a high-precision device. Our original hypothesis was that we could construct a multifunctional sensor with only a single metal deposition on a single substrate. The fabrication methods are extremely important, as we wanted to develop a very inexpensive sensor so that it would have a variety of applications, including home use. Of course, we were also concerned with the resulting accuracy of the sensor, but that was not our primary motivation. For instance, there are many excellent miniaturized pH sensors that have been developed with higher sensitivities than we report here. However, we know of no other sensor at this time that can measure pH, conductivity, and ORP simultaneously, that has as simple a fabrication process, and that has the potential to have a lifetime of months or years. In addition, the technique that we use to measure pH is relatively unique and might be applicable in other situations.

## 2. Materials and Methods

**Figure 1:**
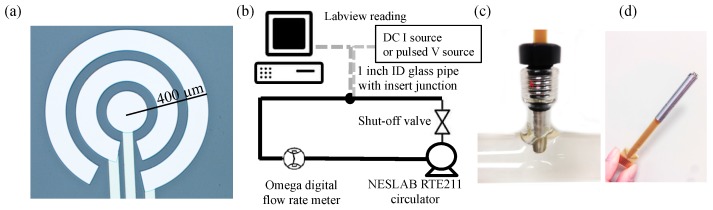
(**a**) Sensor geometry; (**b**) System schematic; (**c**) The sensor inserted into a 1-inch diameter pipe; and (**d**) The assembled sensor probes.

The sensor (Figure 1a) was physical vapor deposited 300/1000 Å Ti/Pt on a glass wafer. The gap between the electrodes was 50 µm. The pressure was controlled under 2 × 10^−6^ Torr with deposition rates of 15 and 5 Å/s. The outside radii of the electrodes were 400 µm, 250 µm, and 100 µm. The sensor was integrated with PC board and inserted into a glass tube connected with a water pump (Figure 1b–d), and the flow rate of the water was controlled at 2.0 gallons per minute (GPM)—the common flow rate in household faucets—for all conductivity, pH, and ORP tests. DI water and chemicals were added into the water to change the properties of the water in both increasing and decreasing directions several times for each experiment. The fluid was in turbulent status ensuring good mixture, and the pH and ORP were monitored in real-time by using YSI 4010-3 Multilab pH and ORP meters to detect pH and ORP without buffer. The conductivity was changed with NaCl, the pH was changed with HCl and NaOH, and the ORP was changed with HClO. The Omega digital flow rate meter was the FP1402 model.

For the conductivity test, a voltage was pulsed at 0.5 V and 6200 Hz on the two larger electrodes on the sensor. The frequency and voltage were chosen based on the work from Laugere et al. [4] to produce a large signal without aliasing or generating interfering reactions. The flow rate of water was controlled at 2.0 GPM. The two outer electrodes were connected in series with an off-chip resistance, *R*, and the root-mean-square current passed through the resistance, *I*rms, was measured as a conductivity indicator.

In pH and ORP tests, the largest electrode was the anode (+), the middle one was the cathode (−), and the smallest electrode was the ORP sensing electrode. The selection was designed to have proper current density on the electrode. The sensor was supplied 0.15 µA by Keithley 2401 sourcemeter, the current flew from the anode to the cathode in the solution, and the voltage differences were measured by Labview 2011. Labview measured the potential differences between the anode and the smallest electrode as ∆V_1_ (>0) and between the cathode and the smallest electrode as ∆V_2_ (<0) simultaneously. ∆V_2_ indicated ORP while the difference (∆V_1_ − ∆V_2_)—which equaled the potential difference between the anode and the cathode—indicated pH. The flow rate of water was also controlled at 2.0 GPM.

## 3. Results and Discussion

**Figure 2:**
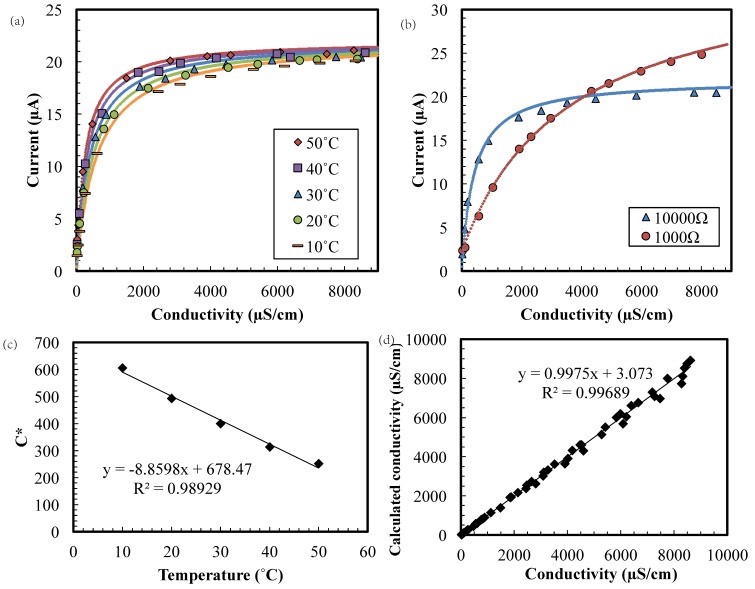
The root-mean-square (rms) current versus conductivity (**a**) in 10–50 °C water with 10 kΩ and (**b**) with 1 kΩ and 10 kΩ in 30 °C. (**c**) The constant C* versus temperature and (**d**) Measurement calculation with Equation (4).

The sensor we have constructed (Figure 1a) can measure three different variables of aqueous solutions: conductivity, pH, and ORP. The performance of the ionic conductivity measurement is shown in Figure 2a. The sensitivity of the measurement can be easily adjusted by changing an off-chip resistance, *R*, connected in series with these electrodes. Figure 2b shows the results of such adjustments, with larger resistances leading to higher sensitivity (i.e., higher d(current)/d(conductivity)) at lower conductivities. For residential water monitoring, conductivities between 1–1000 µS/cm are important because these values are typical of drinking water, and the sensitivity should be about 100 µS/cm. On the other hand, water of 1000–8000 µS/cm is usually found in swimming pools, and the sensitivity in this region should be approximately 1000 µS/cm. Since the lower region is more important in this application, a 10 kΩ resistance was chosen for our experiments.

The conductivity data can be fit with the equation:

(1) $$I_{\mathrm{rms}}=\frac{V_{\mathrm{rms}}}{R+\frac{C_{1}}{\sigma }}=\frac{\sigma \frac{V_{\mathrm{rms}}}{R}}{\sigma +\frac{C_{1}}{R}}=\frac{\sigma I_{\max}}{\sigma +C}$$

This equation is derived from Ohm’s law, and assumes a series resistance established by *R* and the solution conductivity through a cell constant *C*_1_ (unit of length per area, cm^−1^). The saturated current, *I*_max_, can be optimized for a specific range of conductivity by changing *R* in the circuit, as stated previously and shown in Figure 2b.

The influence of water temperature on conductivity measurements shown in Figure 2a can be mitigated by using Equation (2), in which *σ*_0_ is the calibrated conductivity at a given temperature, *T*_0_, and *σ_t_* is the conductivity at temperature *T* [17]:

(2) $$\sigma_{0}=\frac{\sigma_{t}}{1+A\left(T-T_{0}\right)}$$

The combination of Equations (1) and (2) yields

(3) $$I_{\mathrm{rms}}=\frac{\sigma_{t}I_{\max}}{\sigma_{t}+C}=\frac{\sigma_{0}I_{\max}}{\sigma_{0}+\frac{C}{1+A\left(T-T_{0}\right)}}=\frac{\sigma_{0}I_{\max}}{\sigma_{0}+C^{*}\left(T\right)}$$

**Figure 3:**
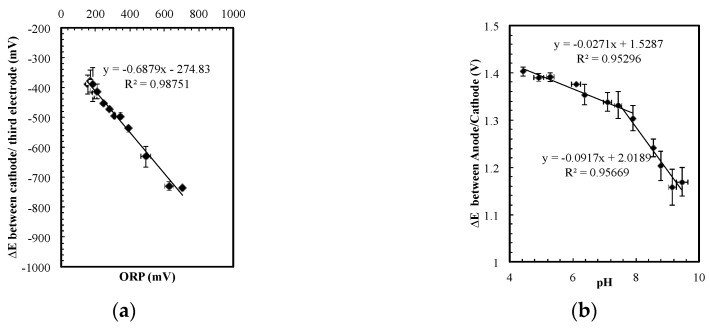
(**a**) The potential difference between the third electrode and the cathode indicates oxidation-reduction-potential (ORP); (**b**) The potential difference between cathode and anode was a pH indicator in various chloride concentrations.

The function C*, plotted in Figure 2c, was derived from Figure 2a with *A* = 2%—a typical temperature coefficient for water. The R^2^ in Figure 3c,d was the coefficient of determination of linear regression. The conductivity calculated using a rearranged version of Equation (3) (i.e., Equation (4)) then agrees very well with the measured conductivity (Figure 2d).

(4) $$\sigma_{0}=\frac{I_{\mathrm{rms}}C^{*}}{I_{\max}-I_{\mathrm{rms}}}$$

The same electrode system can be used to measure the ORP and pH by changing the signal from AC to DC. As shown in Figure 3a, when a 0.15 µA DC current was passed from the largest electrode (anode) to the middle electrode (cathode), the potential difference between the cathode and the third (smallest) electrode, ∆V_2_, indicated the ORP value in the solution. ORP is defined as the open circuit potential between a platinum electrode and a reference electrode offering a stable potential. The linear ORP measurement in Figure 3a suggested not only that the sensor could measure ORP but also that the cathodic potential remained relatively constant in various conditions. In Figure 3, the sensor was operated in a wide conductivity range (200 to 8000 µS/cm) that corresponded to chloride concentrations of 60 to 2700 mg/L, and a wide range of pH values from 4 to 10.

The ORP results suggest that the potential on the cathode remains relatively constant in the wide-ranging conductivity and ORP conditions. This steady cathodic potential implied that the potential difference between the anode and the cathode could be used to indicate pH. The data in Figure 3b was obtained with the sensor operating in the same condition range and at the same time as Figure 3a. Despite the fact that the ORP value and the salt concentration varied, the sensor accurately detected pH changes from 4 to 10. This pH sensor is not extremely precise, but it is sufficient to evaluate pH changes on the order of 0.5 to 1 pH units in this range.

**Figure 4:**
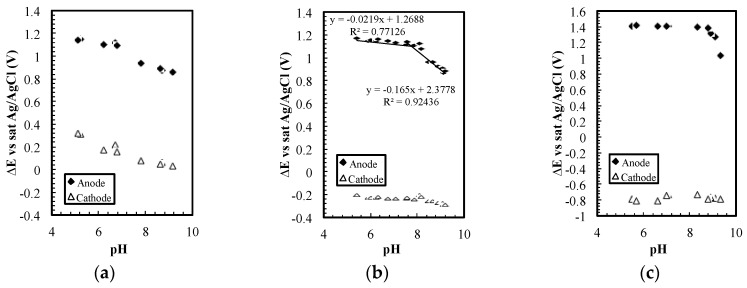
(**a**) The potential on the cathode and the anode versus saturated Ag/AgCl reference electrode at 0.05 µA, (**b**) 0.15 µA, and (**c**) 1.0 µA.

The relatively stable potential on the cathode only appears in a specific current density range. As shown in Figure 4a, the cathode potential changed ~300 mV for 5 pH units at 0.05 µA (~30 µA/cm^2^), while in Figure 4b,c (~90 and 580 µA/cm^2^), the potential changed fewer than 80 mV. If the current density was increased to 33 µA (~200 mA/cm^2^), the dominant reactions should be water electrolysis. Based on the Nernst Equation, the potential on the cathode will change ~300 mV again for 5 pH units.

The electrochemical reactions that produced the stable cathodic voltage presumably started with active sites on the platinum cathode’s surface being occupied by chloride through an adsorption reaction [18]. The reactions are not completely known, but to understand the reaction on the electrodes, the potential changes on the cathode and anode were examined with an additional Ag/AgCl reference electrode. As shown in Figure 4b, the potential on the anode changed with pH, while the potential on the cathode remained relatively stable. The reaction on the cathode was presumably chloride adsorption on platinum surface, as listed in Equation (5) [18]:

(5) $${\mathrm{Pt}}^{*}-\mathrm{Cl}+e^{-}↔{\mathrm{Pt}}^{*}+{\mathrm{Cl}}^{-}$$

If electrolysis of water occurred, hydrogen generation would appear through the cathodic reactions depicted in Equations (6) and (7) [19]:

(6) $$H^{+}+{\mathrm{Pt}}^{*}+e^{-}↔{\mathrm{Pt}}^{*}-H$$

(7) $${\mathrm{Pt}}^{*}-H+H^{+}+e^{-}↔H_{2}+{\mathrm{Pt}}^{*}$$

However, according to the Nernst equation, the potential on the cathode should vary with pH if a half-reaction involves H^+^, or with the log of the concentration if it involves other dissolved ions. Thus, the relative independence of the cathode potential from pH suggests that H^+^ is not involved in the cathodic half-reaction. Further, the supplied current density was ~90 µA/cm^2^ and at least 200 mA/cm^2^ is typically required to drive water electrolysis [20].

Chloride adsorption on platinum has been studied previously, but the value of the half-reaction has been underestimated because it is usually considered a catalyst “poison” in electrochemical reactors such as hydrogen fuel cells. Chloride strongly chemisorbs, forming small polarity bonds on platinum(111) facets [21]; the strength of this bond is essential for Pt/PtCl, providing a relatively stable potential. The half-reaction in Equation (5) was studied in detail by Stern [18], who found that it corresponded to a pH-independent peak in cyclic voltammetry. Chloride adsorption can passivate platinum to both hydrogen and hydroxide ions between 0.3 V and 0.7 V (vs. standard hydrogen electrode, SHE) [22]. The potential required to drive complete chloride desorption was found to be pH and chloride-concentration-independent at sufficiently negative potentials (<−320 mV vs. saturated calomel electrode, SCE) [21]. In addition to a covalent metal/ion interaction, Rose and Benjamin suggested that an adsorbed water layer may solvate the chloride as well [23]. The adsorption of other halide anions on platinum(111) and gold(111) has also been studied in situ by X-ray scattering [24].

The anode potential varied with pH when chloride was present, but remained relatively constant with respect to pH in chloride-free test solutions. Thus, the reaction was suspected to be hypochlorite generation. Hypochlorite dissociates with a pKa of 7.5 [25], which may cause the slope change in pH measurement; the observed cell potentials are consistent with the half-reactions.

(8) $$\mathrm{HClO}+H^{+}+2e^{-}↔{\mathrm{Cl}}^{-}+H_{2}O$$

(9) $${\mathrm{ClO}}^{-}+2H^{+}+2e^{-}↔{\mathrm{Cl}}^{-}+H_{2}O$$

The pH-sensing electrode appears to generate hypochlorite from chloride, which is the most common ion in water reservoirs. It is thermodynamically favorable for oxygen generation to occur through

(10) $$O_{2}+4H^{+}+4e^{-}↔2H_{2}O$$

Although the standard potential of the half-reaction in Equation (8) is 1.49 V [26] and that in Equation (10) is 1.229 V vs. SHE [27], there is a considerable overpotential for oxygen generation on metallic Pt [20,28,29], so hypochlorite generation may be favored kinetically.

The sensor can measure pH and ORP simultaneously with only simple platinum electrodes, but the phenomenon allowing pH and ORP measurement only occurred in a small current density range. As shown in Figure 4b, when 0.15 µA (~90 µA/cm^2^) was used as the driving current, the change in potential on the cathode with respect to pH was smaller than the change on the anode. If the current was decreased to 0.05 µA (~30 µA/cm^2^), potentials on both electrodes changed with pH (Figure 4a) and the potential difference could no longer be used to indicate pH. In Figure 4c, when 1.0 µA (~580 µA/cm^2^) was used, the potential on the anode was not sensitive to pH in the lower pH region. This result suggests that evolution of chlorine or oxygen gas could replace chlorite generation at this current density.

**Figure 5:**
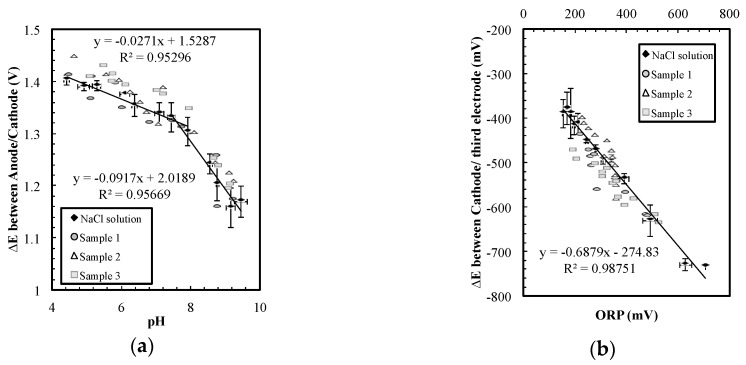
(**a**) The pH measurement and (**b**) the ORP measurement in the water samples.

The sensor presented also showed sufficient tolerance to the interference of other common ions in tap water, and thus the sensor can be a practical method for water monitoring. Ideal monitoring methods for water safety should be affordable end-point measurements that can detect pH changes without frequent maintenance. The convenience of the sensor system is much more important than precision and speed. To test if our sensor could be used for water-quality measurements, three samples containing common ions in different concentrations were prepared for the experiment (Table 1). HClO was titrated into sample 1 and 3 to change the ORP of the solution. Note that the concentration variations between samples (up to 200 mg/L) were 10 times larger than typical situations listed in Table 2 (<15 mg/L). As shown in Figure 5, the sensor can measure pH and ORP simultaneously in these different water samples with only some minor data spread. The NaCl solution was the same data set from Figure 3 that included the common residential water range (sodium and chloride concentrations of 60 to 2700 mg/L). Figure 5 suggests that the sensor can be embedded in drinking water service lines to detect pH decrement accompanied with contaminations. However, more experiments should be performed with actual water sample from various conditions to validate the sensor performance.

## 4. Conclusions

Simple bare platinum electrodes presented in this paper can be used to sense conductivity, ORP, and pH in aqueous solutions when provided the appropriate current density. The sensor was tested in various conductivity, chloride concentration, pH, and ORP range, and was tested with the existence of common ions in residential water. Due to its simple fabrication with a single metal deposition, the device only costs about 10 cents, and flow sensors, temperature sensors, and heaters (for thermal cleaning) can be added onto the chip with little extra cost [30]. Having a small electrode area (<1 mm^2^) and inert platinum electrode surfaces allows the sensor to be easily installed with little maintenance. If needed, a PVC membrane can be added to the sensor to compensate for the influence of other ions for application in extreme condition, such as sanitizing solutions with high concentrations of chlorine (the coated sensor can detect water with HClO up to 12 ppm). The sensor can also be used in microfluidic applications where a small electrode surface is required.

The main use for the pH and ORP sensor reported here is most likely endpoint water analysis where the volume of solution passing over the sensor is relatively high and duration of the sensor is critically important. While not extremely precise, the accuracy when used in home monitoring systems would be sufficient, and the probes would not require frequent attention from users. Given that the pH and major ion concentrations of tap water are generally constant for years at the same local area (e.g., the concentration of major ions in Ann Arbor tap water changed less than 15 ppm over more than 10 years, and the pH of Ann Arbor tap water has remained 9.3 from 2003 to 2015), these sensors should be able to perform well for the detection of contamination in a water supply.

## Figures and Tables

**Table 1:** Ions in the test samples (mg/L).

Ion	Sample 1 (580 µS)	Sample 2 (870 µS)	Sample 3 (1070 µS)
Ca^2+^	30	0.45	15
Mg^2+^	0	20	10
Na^+^	30.6	113	143
K^+^	4	10	0
Cl^−^	112	80	120
CO_3_^2−^	10	0	0
HCO_3_^−^	61	300	300
SO_4_^2−^	72	80	77
NO_3_^−^	12.7	0	0
NH_4_^+^	0	0	1
PO_4_^3−^	0	1	0
HClO	0–0.5	0	0–0.5

**Table 2:** Major ion concentration of Ann Arbor tap water.

Ion	Max C (mg/L)	Min C (mg/L)	∆C (mg/L)	Year
Ca^2+^	32	30	2	2014–2015
Mg^2+^	24	21	3	2014–2015
Na^+^	61.5	52	9.5	2003–2015
K^+^	3.4	3	0.4	2014–2015
Cl^−^	115	112	3	2014–2015
CO_3_^2−^	92.4	77.4	15	2003–2015
SO_4_^2−^	58	56	2	2014–2015
NO_3_^−^	1	0.47	0.53	2003–2015
NH_4_^+^	0.16	0.11	0.05	2003–2015
PO_4_^3−^	0.26	0.24	0.02	2014–2015
